# Complete mitochondrial genome of the Small Salamander in Korea, *Hynobius unisacculus* (Anura: Hynobiidae)

**DOI:** 10.1080/23802359.2019.1710275

**Published:** 2020-01-14

**Authors:** Jae-I. Moon, Kyo-Soung Koo, Mi-Ae Jeon, Jae-Hyeok Choi, Ha-Cheol Seong, Dong-Hyun Lee

**Affiliations:** a School of Biological Sciences and Biotechnology, College of Natural Sciences, Chonnam National University, Gwangju, the Republic of Korea;; b Department of Biological Sciences, College of Natural Sciences and Research Center of Ecomimetics, Chonnam National University, Gwangju, the Republic of Korea

**Keywords:** *Hynobius unisacculus*, Hynobiidae, mitochondrial genome

## Abstract

The complete mitochondrial (mt) genome of *Hynobius unisacculus* was sequenced and characterized. The circular mt genome constituted of 37 genes (13 protein-coding genes, 22 transfer RNAs, and 2 ribosomal RNAs) and a non-coding region (NCR). Phylogenetic analysis based on the full mt genome sequences confirmed that *H. unisacculus* was closely related to *Hynobius leechii* rather than other *Hynobius* species. This is the first completed mt genome from *H. unisacculus*, which provides data for further study of phylogeny in Hynobiidaes.

The *Hynobius* is a genus of Asian salamander in the family Hynobiidae, currently including 38 species and endemic to Korea, Japan, China, and Russia (Weisrock et al. 2013; Sugawara et al. 2018). Four salamander species in *Hynobius* genus have been reported in Korean Peninsula: *Hynobius leechii*, *Hynobius yangi*, *Hynobius quelpaertensis*, and *Hynobius unisacculus* (Baek et al. 2011; Lee et al. 2011; Min et al. 2016). Among these salamander species, Small Salamanders (*H. unisacculus*) were reported in 2016 as a new species in *Hynobius* genus, which dominantly inhabit the southeast coast of Jeollanam-do, and are related to other three species morphologically and genetically (Min et al. 2016). While the full sequences of mitochondrial (mt) genome from other three salamander species in Korean Peninsula were previously reported in NCBI (Figure 1), the complete mt genome of *H. unisacculus* has not been identified and also, the ecological and biological importance of this species has been poorly understood. Here, we sequenced the full mt genome of the *H. unisacculus*, which can help its phylogenetic position and evolution of genomes.

**Figure 1.**
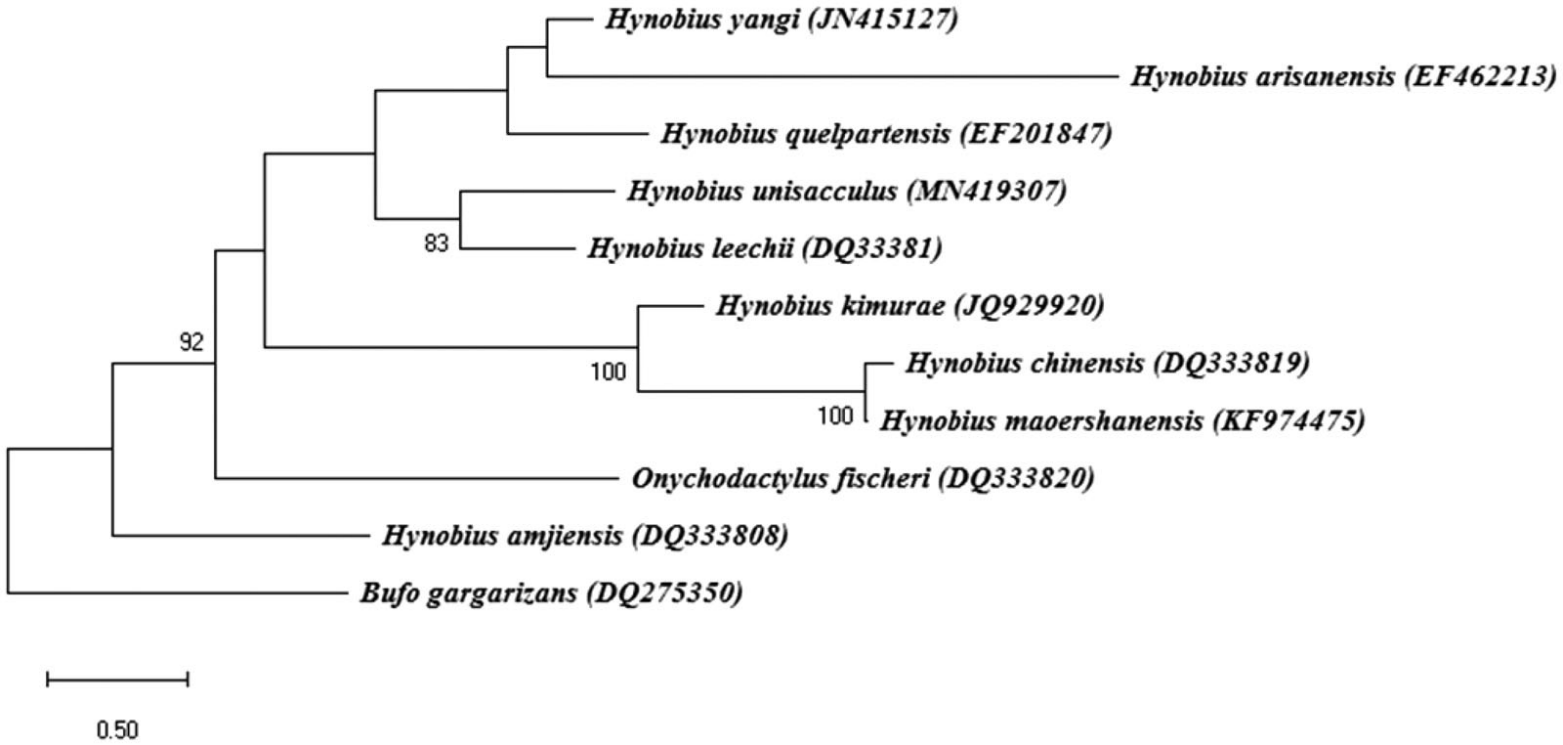
Phylogenetic tree of *Hynobius unisacculus* and other related species based on complete mitochondrial (mt) genome data. The bootstrap value based on 1000 replicates is shown on each node. *Bufo gargarizans* was used as the outgroup for tree rooting. The phylogenetic analysis was performed using MEGA7 (Saitou and Nei 1987).

The *H. unisacculus* specimen was collected from the southern coast of Korea (34°27′3.45′′N, 127°29′42.94′′E). We extracted the genomic DNA from the subsample (muscle) using the DNeasy Blood & Tissue kit (Qiagen, Valencia, CA) according to the manufacturer’s protocol and the extracted DNA sample was deposited at the Museum of Wildlife, located in Research Center of Ecomimetics, Chonnam National University (Specimen accession number: 2019-RCE-HU021). We determined the complete mt genome sequence using the next-generation sequencing reads (400 bp length in each read) generated from MiSeq (Macrogen, Seoul, Korea). Mapped reads were used for *de novo* assembly and annotation by using commercial software (MITOS, http://mitos.bioinf.uni-leipzig.de/index.py) to identify the full mt genome with about an average 150 × coverage.

The complete mt genome of *H. unisacculus* was 16,413 bp in length deposited in GenBank (Accession No. MN419307) and contains 13 protein-coding genes (PCGs), 22 transfer RNA (tRNA) genes, 2 ribosomal RNA genes (*srRNA* and *lrRNA*), and a putative long non-coding control region (NCR). 12 protein-coding genes, 14 tRNAs, and 2 rRNAs were predicted to be transcribed from the same strand (heavy strand), whereas 1 protein-coding gene (NADH dehydrogenase subunit 6) and 8 tRNA genes (*tRNA^Gln^*, *tRNA^Ala^*, *tRNA^Asn^*, *tRNA^Cys^*, *tRNA^Tyr^*, *tRNA^Ser^*, *tRNA^Pro^*, and *tRNA^Glu^*) were encoded on the light strand. The nucleotide composition of the *H. unisacculus* (A = 32.0%, C = 20.7%, G = 33.5%, and T = 13.8%) was similar to that of *H. leechii* mt genome (A = 32.1%, C = 20.6%, G = 33.5%, and T = 13.7%), *H. yangi* (A = 32.4%, C = 20.4%, G = 33.6%, and T = 13.5%), and *H. quelpartensis* (A = 32.4%, C = 20.5%, G = 33.2%, and T = 13.9%). The sequence comparisons between *H. unisacculus* and *H. leechii* indicated a 94.5% sequence identity, and place *H. yangi* and *H. quelpartensis* as sisters to these two species (Figure 1).

In order to investigate the phylogenetic position of *H. unisacculus*, the full mt genome sequences of 10 salamander species were extracted from Genbank, and *Bufo gargarizans* served as outgroup. While a previous study on phylogenetic analysis with partial mt genome of *H. unisacculus* (Min et al. 2016), where only 1607 bp of mt genome containing two mitochondrial genes (*cyt b* and *12S rRNA*) were compared, showed that *H. quelpartensis* was the most closely related to *H. unisacculus*, phylogenetic analysis based on the full mt genome sequences demonstrated that *H. unisacculus* was clustered in a monophyletic group with *H. leechii* (Family Hynobiidae), and also share close relationships with other salamander species, including *H. yangi* and *H. quelpartensis* (Figure 1). These data provide important molecular information for further evolutionary analysis of the phylogenetic relationships of the family and also acts as a useful genetic marker for identification and ecological studies on Hynobiidaes.
